# Can prebiotics help tackle the childhood obesity epidemic?

**DOI:** 10.3389/fendo.2023.1178155

**Published:** 2023-05-26

**Authors:** Yaqin Wang, Anne Salonen, Ching Jian

**Affiliations:** ^1^ School of Life and Health Technology, Dongguan University of Technology, Dongguan, China; ^2^ Department of Food and Nutrition, University of Helsinki, Helsinki, Finland; ^3^ Human Microbiome Research Program, Faculty of Medicine, University of Helsinki, Helsinki, Finland

**Keywords:** prebiotics (Sources: MeSH), childhood obesity, gut microbiota, short-chain fatty acids (SCFAs), bacteroides

## Abstract

Globally, excess weight during childhood and adolescence has become a public health crisis with limited treatment options. Emerging evidence suggesting the involvement of gut microbial dysbiosis in obesity instills hope that targeting the gut microbiota could help prevent or treat obesity. In pre-clinical models and adults, prebiotic consumption has been shown to reduce adiposity partially *via* restoring symbiosis. However, there is a dearth of clinical research into its potential metabolic benefits in the pediatric population. Here, we provide a succinct overview of the common characteristics of the gut microbiota in childhood obesity and mechanisms of action of prebiotics conferring metabolic benefits. We then summarize available clinical trials in children with overweight or obesity investigating the effects of prebiotics on weight management. This review highlights several controversial aspects in the microbiota-dependent mechanisms by which prebiotics are thought to affect host metabolism that warrant future investigation in order to design efficacious interventions for pediatric obesity.

## Introduction

Excess weight during childhood and adolescence remains one of the most challenging issues in global health. In Europe, overweight and obesity currently affect more than one quarter of children (29% of boys and 27% of girls) (1). The global rate of overweight increased from 10.3% in 2000 to 18.4% in 2018, especially in the 5-19 age group (2). This surge in childhood overweight and obesity is more severe in low-income and middle-income countries over the past four decades (3), and has been exacerbated by the COVID-19 pandemic (4). Approximately three-quarters of children with overweight or obesity carry the weight status into adulthood, and therefore childhood obesity increases both immediate and imminent co-morbidity risks for several non-communicable diseases, such as cardiovascular diseases, non-alcoholic fatty liver disease (NAFLD), diabetes, cancer, and some immune-related disorders (5).

The etiology of obesity involves complex interactions between a compendium of environmental and genetic factors that result in an energy imbalance, leading to the deposition of excess adipose tissue. While genome wide association studies have identified several gene loci associated with obesity, genetic susceptibility accounts for only a small percentage of the variance in body weight (6, 7). Importantly, the rapid rise in obesity rates in recent years cannot be attributed to any genetic changes occurring on such short evolutionary time frame, suggesting that environmental factors such as diet and levels of physical activity play a larger role (8). As a metabolic gateway between the outer environment and the host (9), the gut microbiota (i.e., the trillions of microorganisms residing within the human gastrointestinal (GI) tract) has come under the spotlight for its potential contribution to the childhood obesity epidemic (10).

Current treatment strategies for childhood obesity center around lifestyle counseling with multicomponent approaches based on dietary changes, behavior therapy, physical activity, and to a lesser extent, pharmacotherapies, with limited success (4, 11, 12). Some evidence suggests that interventions that only focus on diet are less effective in older children (12). In general, lifestyle changes are difficult to implement over the long term. Although bariatric surgery is the most effective and durable treatment for inducing weight loss in adolescents with severe obesity, it is rarely performed in the pediatric/adolescent population (ca. less than 1% of all weight loss procedures) due to the perceived invasiveness, complications and irreversibility of the procedures as well as high costs (4). Consequently, research efforts have focused on identifying alternative strategies for long-term prevention and treatment of childhood obesity. Prebiotics, a term first coined in 1995 and currently rereferred to as “substrates that can be selectively utilized by host microorganisms conferring health benefits” (13), have been increasingly touted as one of such alternative approaches.

The definition and mechanisms of action of prebiotics (reviewed elsewhere in detail (13–15)) partly overlap with that of dietary fiber. The main difference is that prebiotics selectively stimulate certain commensals, while not all fibers show prebiotic properties. Current prebiotics are predominantly carbohydrate-based, with fructans (e.g., inulin and fructooligosaccharides (FOS)) and galactooligosaccharides (GOS) produced from lactose being the most common prebiotics studied to date (15). Although evidence from animal models suggests beneficial effects of prebiotics on metabolic parameters related to obesity (16), few clinical studies have been conducted in the pediatric population. In this article, we summarize the available clinical trials on childhood obesity using prebiotics, focusing on the 5-19 age group. We also highlight some controversial aspects in the microbiota-dependent mechanisms by which prebiotics are thought to affect host metabolism and discuss how prebiotics can help reduce the burden of childhood obesity.

## Features of the gut microbiota in childhood obesity

Over the past two decades, differences in gut microbiota composition and its metabolic activity between individuals with and without obesity have been reported (17, 18). Through investigation into these transmissible and rapidly modifiable features of the gut microbiota, multiple mechanisms for the link between obesity and the gut microbiota have been proposed, including increased energy extraction from the diet, regulation of satiety and other gut hormones e.g., through activation of enteroendocrine signaling pathways by short-chain fatty acids (SCFAs; acetate, propionate and butyrate) and other microbial metabolites, modulation of glucose and energy homeostasis through bile acid and other signaling, and increased local and systemic inflammation caused by a variety of microbially derived molecules especially lipopolysaccharides (LPS) (19, 20). SCFAs produced through colonic fermentation of non-digestible carbohydrates have represented a focal point in the obesity-microbiome research field owing to their seemingly contrasting properties i.e., acting as an energy source as well as a diverse signaling moiety (21). The latter modulates intestinal barrier function, glucose homeostasis, lipid metabolism, appetite, and immune function (21, 22). Children and adults with obesity appear to have higher fecal concentrations of SCFAs compared to lean controls (23), suggestive of increased production. It should be noted that gut epithelia have the capacity to absorb up to 95% of SCFA before excretion (21). Therefore, the SCFA concentrations measured from feces are prone to variation, since SCFA absorption efficiency depends on the type of substrate and site of fermentation (more time to absorb for readily fermented substrates in the proximal than distal colon) and also likely differs between individuals (24, 25). Moreover, the contribution of colonic fermentation to daily energy supplies, initially calculated to be up to 10% (26), is likely an overestimation. By depleting the gut microbiota using calorie restriction and antibiotic treatment in healthy volunteers, Basolo et al. recently estimated that the gut microbiota harvests an additional ~2.5% of absolute ingested calories that would translate to a mere 1.2-kg weight gain in a 100-kg subject over one year (27). In this regard, it remains unclear to what extent the energy extraction capacity of the microbiota differs between individuals with obesity and those who are lean.

A recent systematic review of case-control studies comparing the gut microbiota of individuals with and without obesity concluded that LPS-producing Proteobacteria was the most consistently reported obesity-associated phylum, and *Faecalibacterium*, *Akkermansia*, and *Alistipes* as lean-associated genera in both adults and children (17). However, in childhood obesity discrepancies were observed for several bacterial taxa, notably the genus *Bifidobacterium* (17), likely reflecting the transition of *Bifidobacterium* species and dominance in children toward adulthood (28). Hence, the higher heterogenicity of the gut microbiota features in childhood obesity compared to that in adults may result from a synergic effect of age and obesity on the gut microbiota (17, 29). To date, no studies have employed metagenomic sequencing that could unravel specific bacterial species, strains and functions as well as potential viruses or fungi specifically involved in the development of pediatric obesity in a sufficiently large cohort (10, 17).

**Figure 1:**
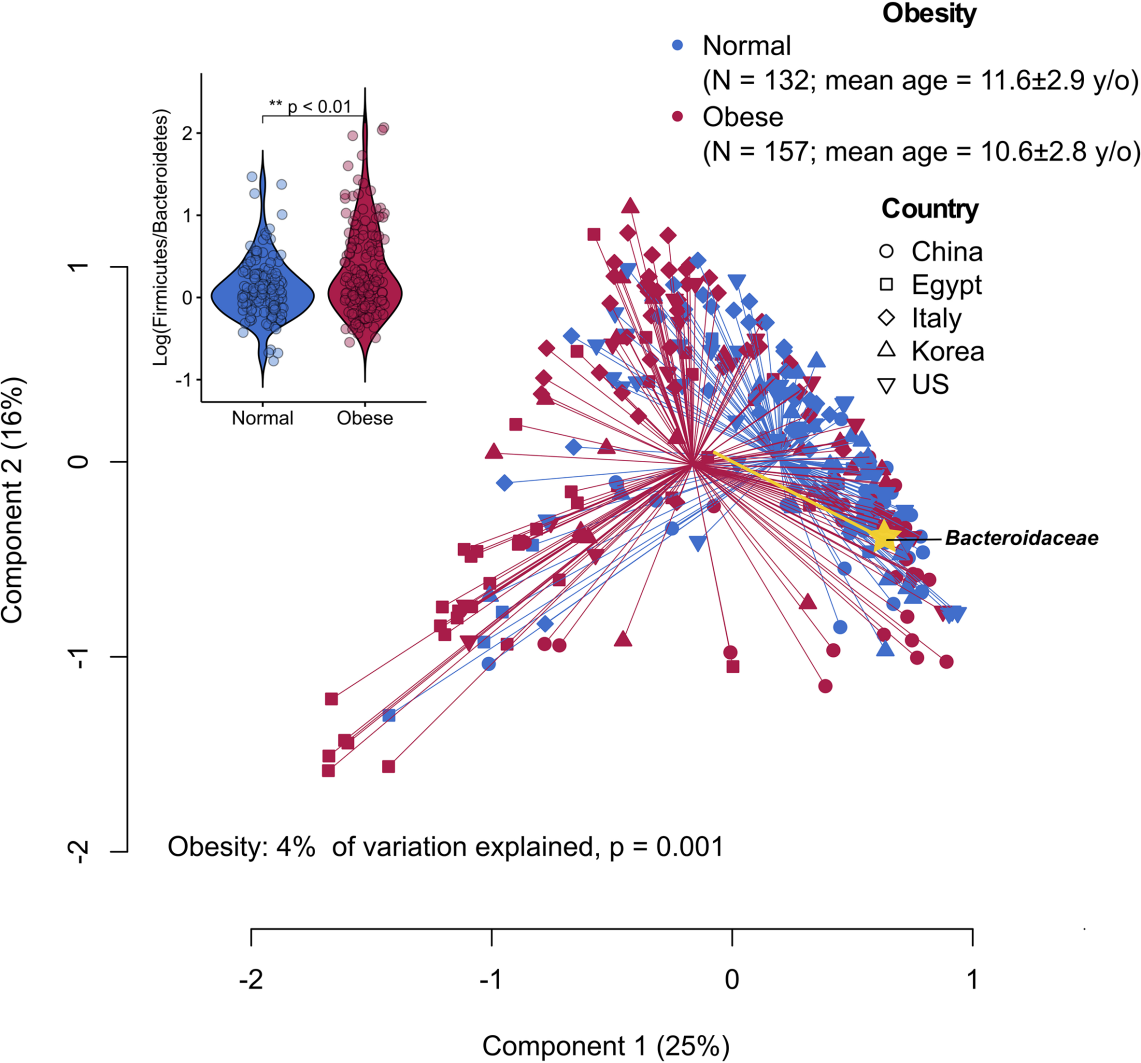
Principal coordinate analysis (PCoA) plot of family-level microbiota variation based on the Bray-Curtis dissimilarity matrix from five publicly available datasets of childhood obesity generated by comparable sequencing technology (PRJCA002952, PRJNA637782, PRJNA647465, PRJNA774503, PRJNA317290). Raw read sequence extraction, filtering and processing were performed as described previously (33). The processed sequences were analyzed at the family level that can capture most of the information while avoiding potential between-study technical variation (34). Obesity status explained 4% of the microbiota variation (P = 0.001, permutational multivariate analysis of variance). Yellow biplot indicates *Bacteroidaceae* that drives sample clustering on the plot. Box plot at the upper-left corner shows the logarithmic values of the Firmicutes-to-Bacteroidetes ratio from all samples according to obesity status.

The ratio between the two predominant bacterial phyla, Firmicutes and Bacteroidetes, represents some of the most striking cross-study discordance in microbiota features of obesity (17, 30, 31). The discrepant findings may be due to differences in research methods and cohort-specific characteristics (e.g., ethnicity and diet) (32). By re-analyzing publicly available sequencing datasets derived from case-control studies using a uniform bioinformatic pipeline, the gut microbiota of children with obesity appeared to have an increased ratio of Firmicutes to Bacteroidetes primarily driven by the reduced relative abundance of *Bacteroidaceae* (**Figure 1**), suggesting common causal agents in the microbiota and/or similar physiological states in the intestinal milieu across continents and cultures. Of note, various *Bacteroides* spp. (dominant taxa in the family *Bacteroidaceae*) have been shown to inhibit or alleviate metabolic dysregulation by activating folate-mediated signaling pathways (35), reducing production of pro-inflammatory LPS (36) and modulating circulating amino acids (37, 38). On the other hand, *Bacteroides* spp. with the bile salt hydrolase (BSH) activity selectively hydrolyze conjugated bile acid substrates to alter bile acid compositions, resulting in worsened metabolic phenotypes in animal models (39).

Taken together, the altered gut microbial community in obesity and the evidence suggesting its connection to early-life perturbations (10) instill hope that targeting these disruptions could help prevent or treat childhood obesity. Nevertheless, observational studies, especially those employing shotgun metagenomic sequencing, on the associations between the gut microbiota and childhood obesity remain instrumental in understanding what caused the cross-study inconsistencies and identifying relevant targets and timing for rationally designed interventions. For example, few longitudinal studies in the pediatric population have examined the time of obesity onset and development of an obese-like gut microbiota, which would inform the right timing for potential interventions.

## Potential mechanisms of action of prebiotics in metabolic improvements

**Figure 2:**
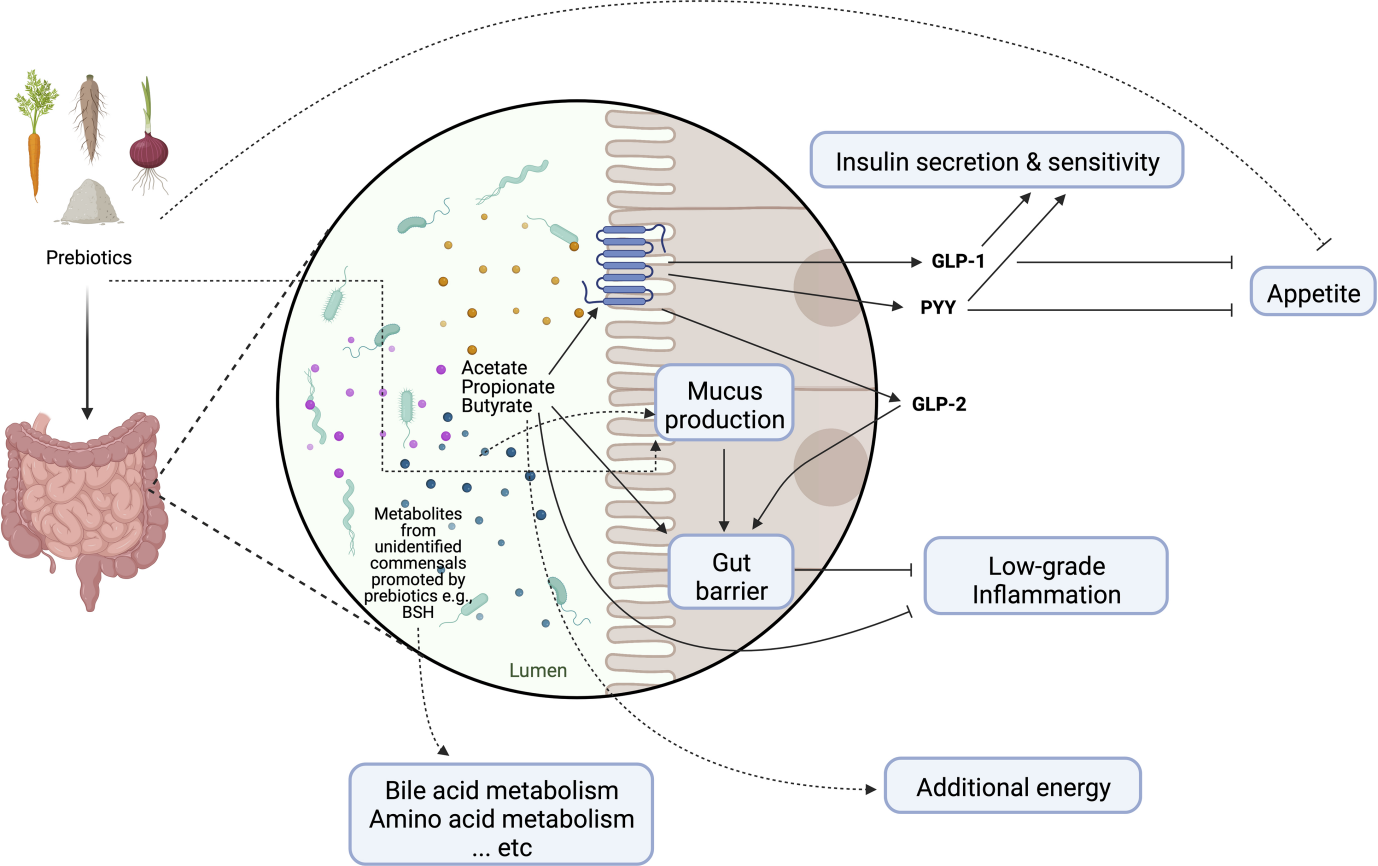
Schematic representation of main mechanisms of actions through which prebiotics may influence host metabolism. Dotted lines represent purported mechanisms under investigation. GLP-1, glucagon-like peptide-1; GLP-2, glucagon-like peptide-2; PYY, peptide YY; BSH, bile salt hydrolase. Created with Biorender.com.

The modulation of the enteroendocrine function by prebiotics and their fermentation products is often cited as the main mechanism behind the systemic effects on lipid and glucose homeostasis, as well as on satiety control (**Figure 2**) (16). For instance, circulating levels of glucagon like peptide-1 (GLP-1) and anorexigenic peptide YY (PYY) have been shown to be elevated by SCFAs following prebiotic supplementation in both mice and humans (16). Moreover, early murine studies suggest that the prebiotic-induced proliferation of commensal bacteria altered the mucosal architecture (40). The altered mucus composition and/or regular turnover of mucin glycoproteins may help maintain the integrity of the mucosal barrier and attenuate inflammation in the context of obesity (41, 42). It should be note that some prebiotics may directly interact with the host to promote barrier function and improve glucose metabolism, the identification of which from metabolic studies in humans is nevertheless extremely difficult (13). The prebiotic effect may be further enhanced by increasing the endogenous production of intestinal glucagon like peptide-2 (GLP-2), which is known to reinforce gut barrier function through upregulation of critical tight junction proteins in the epithelium (43). Since several above-mentioned changes coincided with an increase in fecal bifidobacteria, the health-promoting effects of prebiotics have been attributed mainly to this bacterial population group. However, the role of bifidobacteria in mediating prebiotic-induced metabolic improvements has been questioned in some studies (44). Importantly, what constitutes a healthy gut microbial community is often age and/or population-dependent (45); it is now recognized that prebiotic effects extend beyond bifidobacteria and would likely require a consortium of gut microbes involved in the trophic interactions (14). In addition to the microbiota-dependent mechanisms, prebiotics also contribute to increased dietary fiber intake and decreased energy density of the diet that has been associated with reduced odds of visceral obesity in overweight adolescents (46).

## Clinical evidence for prebiotics as a dietary intervention in childhood obesity

As summarized in **Table 1**, the few clinical studies in children with overweight or obesity to date showed no consistent effects of prebiotics on weight management or insulin sensitivity. Most of the studies empirically used inulin-type fructans (ITFs) as the choice of prebiotic supplementation likely based on their relatively well-studied metabolic benefits in adults (56, 57). Zalewski and Szajewska investigated the effect of glucomannan (a viscous dietary fiber derived from the plant *Amorphophallus konjac* with a bifidogenic effect (58)) and found decreased levels of total and low-density lipoprotein cholesterol with no body weight reduction after 12 weeks of supplementation, though the gut microbiota was not analyzed (53). Additionally, one ongoing trial is studying the effect of polylactose, a newly synthesized prebiotic derived from lactose, on the liver fat in children with obesity and NAFLD (see www.clinicaltrials.gov NCT04100109).

**Table 1:** Summary of clinical trials with prebiotics in children with overweight or simple and genetic obesity.

Study	Prebiotic	Study population and design	Gut microbiota	Inflammation	Entero-endocrine	Others	Adiposity, lipid and glucose metabolism	Ref.
Abrams et al. (2007)	Oligofructose-enriched inulin	US; 9-13y (N=97); 8 g/d for 48 w	N.D.	N.D.	N.D.	Increased accretion of calcium to the skeleton	Smaller BMI increase	(47, 48)
Liber and Szajewska (2014)	Oligofructose	Poland; 7-18y (N=97); 8 g/d of for 7–11 y and 15 g/d for 12-18y for 12w with dietary advice and PA	N.D.	N.D.	N.D.	N.D.	No effect on body weight	(49)
Zhang et al. (2015)	Mixed prebiotics	China; 3-16y (N=38), grouped by simple obesity (SO) or Prader-Willi syndrome (PWS)); whole meal replacement with prebiotics for 4w (SO) or 12w (PWS)	Increased *Bifidobacterium* spp. and enhanced carbohydrate metabolism	Reduced inflammation	Reduced leptin and increased adiponectin	Reduced urine levels of TMAO, indoxyl sulfate, phenylacetylglutamine and hippurate	Reduced BMI, improved liver condition, lipid and glucose metabolism	(50)
Hume et al. (2017), Nicolucci et al. (2017)	Oligofructose-enriched inulin	Canada; 7-12y (N=42); 8 g/d of prebiotic for 16w	Increases *Bifidobacterium* and reduced *Bacteroides vulgatus*	Reduced inflammation	Increased fasting ghrelin and adiponectin	Increased satiety and levels of primary bile acids	Reduced BMI z-score, body fat and trunk fat, serum triglycerides	(51, 52)
Zalewski and Szajewska (2019)	Glucomannan	Poland; 6-17y (N=81); 3 g/d of glucomannan for 12w with dietary advice and PA	N.D.	N.D.	N.D.	N.D.	No effect on weight reduction; improved lipid metabolism	(53)
Visuthranukul et al. (2022)	Oligofructose-enriched inulin	Thailand; 7-15y (N=155); 13 g/d of prebiotic for 24w with dietary advice and PA	N.A.	No difference between study groups	N.D.	N.D.	Increased fat-free mass index	(54, 55)

PA, physical activity; BMI, body mass index; TMAO, trimethylamine n-oxide; N.D., not determined; N.A., not available.

Currently, only two clinical trials on prebiotic supplementation in pediatric obesity, both reporting positive outcomes in reducing inflammatory markers as well as BMI, assessed gut microbiota composition (50, 51). The two studies similarly reported an increase in bifidobacteria in the gut microbiota after the intervention (50, 51), but the increase appeared not to correlate with changes in metabolic or inflammatory markers (51). Instead, the reduction in *Bacteroides vulgatus*, a bacterial group linked to both positive (36, 37, 59, 60) and negative (61, 62) metabolic health previously, was associated with a reduction in percent trunk fat over the 16-week intervention (51). Notably, there is no data on the long-terms effects of prebiotics beyond one year, which may have been required for some microbiota-dependent mechanisms to appreciably affect body weight (27). Moreover, puberty can act as a confounder in pediatric clinical trials both in terms of weight development and the gut microbiota (63). Thus, there exists a need for clinical trials assessing long-term changes in metabolic, enteroendocrine and inflammatory markers as well as in the gut microbiota with the use of rationally designed/selected prebiotics in the pediatric population.

## Discussion and perspectives

Unlike rodent models of obesity with a salient and transferrable microbiota component in the pathophysiology (64), in humans controversies exist regarding whether the difference between the gut microbiota of individuals with and without obesity is the cause or effect of altered diet and energy metabolism and, if the former, how much it influences pathophysiology. Despite this incomplete understanding, clinical trials have tested empirically numerous microbiota-targeting therapies, including prebiotics, to prevent or treat obesity with mixed results. Below, we discuss some important aspects of prebiotics that warrant future investigation in order to design efficacious interventions for pediatric obesity.

Human studies in pediatric obesity to date have predominantly used ITFs as the sole prebiotic supplement, which cannot correct the reduced relative abundance of *Bacteroides* spp. in the gut microbiota, characteristic in and potentially mechanistically linked to pediatric obesity as described previously. Recent studies have shown some dietary fibers, such as seaweed polysaccharides and yeast mannan, to selectively promote specific *Bacteroides* species (65, 66). By rationally designing prebiotic fibers to increase the abundances of targeted *Bacteroides*, it was found that prebiotic supplementation significantly altered plasma proteomes linked to microbiota changes in adults with overweight and obesity in a 3-week pilot trial (67). On the other hand, the degree of polymerization (DP) of prebiotics has a significant impact on their fermentation location in the colon; low (e.g., FOS) and high (e.g., inulin) DP fructans tend to be fermented more in the proximal and distal colon, respectively (68). This difference may influence the efficacy of prebiotics since distal SCFA infusion *in vivo* has been shown to induce more pronounced effects on biomarkers than proximal (69).

Administration of prebiotic interventions within the right window of time is critical, as the microbiota of children continues to develop and has higher interindividual variability compared to that of adults. While the optimal timing remains to be determined, it is likely that early interventions would exert a more pronounced effect similar to what was observed in other diet-based interventions for pediatric obesity (12). Although not covered in the scope of this article, human milk oligosaccharides (HMOs) abundant in breastmilk may possess anti-obesogenic properties partly through their prebiotic potential and have been formulated in some commercially available infant formula (70). However, the data on such supplementation, especially regarding its long-term effects, is currently lacking. Specific types of HMOs have been positively associated with child height and weight z scores in a recent cohort study (71), suggesting its growth-promoting effects. Thus, at least theoretically, if HMO content is a poor match to the age-depended needs, it could potentially promote overly fast growth or accumulation of fat mass over fat-free mass (71). On the other hand, animal studies using prebiotics found that prenatal supplementation successfully decreased the offspring’s obesity risk (72), likely *via* the effect of maternal SCFAs that are constantly supplied to the fetus (73). This raises the possibility of intervening an intergenerational cycle of obesity through maternal interventions. Finally, an underexplored area in the use of prebiotic supplementation is the correction of antibiotic-associated dysbiosis, which has been linked to increased childhood BMI (74). In a rat model, increased fat mass, hyperinsulinemia and insulin resistance following direct pulsed administration of antibiotics in early life were prevented by prebiotic co-administration in a sex-specific manner (75).

Overall, taking into consideration the interindividual and inter-population variability in microbiota responses to prebiotics is important to maximize benefits while avoiding potential harms. The variable metabolic improvements in adults with obesity in response to inulin supplementation have been demonstrated to depend on the pre-intervention gut microbiota (76). Holmes et al. showed substantial interindividual variation in the prebiotic effects on SCFA production from the microbiota of adolescents with obesity, which could contribute to the inconsistent results from prior studies of prebiotics in pediatric obesity (24). Thus, therapeutic efforts involving prebiotics in children with obesity may benefit from *a priori* patient stratification based on the gut microbiota. In the global context, one challenge to translating potential clinical benefits of prebiotics is the “uncharacterized” gut microbiota in the populations of developing countries where the rise of pediatric obesity is most alarming. For instance, Egyptian and American adolescents differed considerably in microbiota composition, functions and microbial metabolites (77). This inter-population difference in the gut microbiota may represent a limiting factor for successful prebiotic interventions in the context of pediatric undernutrition. Specifically, rice bran supplementation significantly improved the weight for age z-scores in the infants from Mali, where rice is the dominant staple food, compared to that of Nicaraguan infants who had different gut microbiota and metabolome composition (78). On the other hand, gastrointestinal tolerance differs with the type and amount of prebiotics consumed and is affected by the highly individualized response (79). For example, most of the highly fermentable ITFs may be tolerated in the generally healthy adult population at daily intakes of up to 15 g, depending on their DP (80). In contrast, resistant starches and soluble corn fiber have been consumed at the dose of 50 g/d without symptoms (81). Early studies suggest that infants and young children tolerate ITFs up to 0.8g/kg body weight per day (82), which has been corroborated by the interventions discussed herein (**Table 1**). The gastrointestinal tolerance of other types of prebiotics in children is less documented and warrants formal evaluation in interventional trials (79). Last but not least, prolonged consumption of inulin fiber enriched foods unexpectedly led to potentially dysregulated bile acid and/or SCFA metabolism in recent murine studies (83, 84), contributing to type 2 inflammation and increasing the risk of hepatocellular carcinoma in a microbiota-dependent manner. Although the translatability of these animal models to humans remains unclear, the findings emphasize the need to rigorously report safety data in prebiotic trials conducted in various populations and ethnic groups with different gut microbiota composition. Many of the concerns may also be circumvented by rationally designing locally sourced dietary approaches to target the gut microbiota.

It has been shown that the variability of glycemic response to exercise in prediabetic adults depended on the gut microbiota *via* SCFA production and amino acid catabolism (85). Similarly, prebiotic supplementation and physical activity appeared to have a synergistic effect on BMI reduction in adults with obesity (86). While a recent study in children with obesity argued that the effects of inulin supplementation on body weight and adiposity reduction may be overshadowed by the intensive behavioral modification (55), it will be interesting to see whether prebiotic supplementation provides benefits to those who are the most refractory to exercise-induced weight loss.

Prebiotic fibers in appropriate concentrations improve the textural and sensory qualities of foods, primarily due to their moisture retention capacity and potential off-flavor masking properties (87). Inulin, for instance, has been formulated in sausages and cheese as a fat substitute (88, 89). In light of the recent reports linking certain food additives to metabolic dysregulation *via* dysbiosis (90, 91) as well as the growing clean-label trend, several types of exopolysaccharides naturally synthesized by lactic acid bacteria during fermentation have been proposed as substitutes for flavorants and emulsifiers used in bakery and dairy products (92). Many of these exopolysaccharides have shown potential prebiotic properties in pre-clinical and clinical studies (92). Taken together, the benefit of prebiotic-enriched foods in combatting childhood obesity is twofold. On one hand, prebiotic addition increases dietary fiber content and promotes growth of beneficial commensals. On the other hand, prebiotics also enhance physiochemical properties of the surrounding food matrix, allowing for reduction in fat and food additives as well as energy content without considerably compromising product taste and texture. These benefits can potentially bolster an overall improved sensory experience for the children, thereby promoting long-term adherence to a healthier diet. Importantly, it has been proposed that addition of 2.5-6.5 g prebiotics per serving in food may serve to double or triple one’s regular fiber intake and meet a threshold for beneficial effects with a single dietary modification (93).

## Conclusions

While the available studies in the pediatric population generated mixed results potentially mired by interindividual variation, prebiotic supplementation has shown potential as an adjunctive therapy for improving several metabolic markers when rationally applied as well as reducing energy density of the diet. Their long-term effects and the mechanistic role of the gut microbiota in mediating the potential benefits need to be clarified in more clinical trials directly targeting childhood obesity.

## Author contributions

YW and CJ conceptualized, reviewed the literature, visualized the data, and wrote the manuscript. AS critically revised the manuscript. All authors contributed to the article and approved the submitted version.
